# Permanent Canine Impaction: A Systematic Review of Incidence, Distribution, and Etiology

**DOI:** 10.3390/medicina62040681

**Published:** 2026-04-02

**Authors:** Marina Antoneta Pop, Sorana Maria Bucur, Anca Porumb

**Affiliations:** 1 Doctoral School of Biomedical Sciences, University of Oradea, 1 Universității Street, 410087 Oradea, Romania; burynell23@yahoo.com (M.A.P.); anca.porumb@uoradea.ro (A.P.); 2 Department of Dentistry, Faculty of Medicine, Dimitrie Cantemir University of Târgu Mureș, 3-5 Bodoni Sandor Street, 540545 Târgu Mureș, Romania; 3 Department of Dentistry, Faculty of Medicine and Pharmacy, University of Oradea, 4 Universității Street, 410087 Oradea, Romania

**Keywords:** canine impaction, dental anomalies, maxillary canine, transmigration, root resorption, CBCT, prevalence, etiological factors

## Abstract

*Background and Objectives*: Tooth impaction is a common developmental dental anomaly characterized by the failure of eruption within the expected physiological timeframe. Permanent canines represent the second most frequently impacted teeth after third molars and may lead to functional, esthetic, and orthodontic complications. This systematic review aimed to synthesize current evidence regarding the incidence, anatomical distribution, etiological determinants, and diagnostic evaluation of permanent canine impaction. *Materials and Methods*: A systematic literature search was conducted in PubMed, PubMed Central, and ScienceDirect for studies published between December 2009 and December 2025. Studies reporting prevalence data, anatomical positioning, etiological factors, or imaging characteristics of permanent canine impaction were included. Study selection followed PRISMA 2020 guidelines, and 31 studies were included in the qualitative synthesis. Two independent reviewers screened titles, abstracts, and full texts. Methodological quality was assessed using the Joanna Briggs Institute Critical Appraisal Tools. *Results*: Thirty-one studies met the inclusion criteria and were included in the qualitative synthesis. The reported prevalence of maxillary canine impaction ranged from 0.97% to 7.10%, while mandibular impaction occurred less frequently. Palatal displacement represented the most common positional pattern. Major etiological factors included retained deciduous canines, dental arch constriction, supernumerary teeth, odontomas, and genetic anomalies such as lateral incisor agenesis. Cone-Beam Computed Tomography (CBCT) demonstrated superior diagnostic accuracy compared with panoramic radiography. *Conclusions*: Permanent canine impaction is a multifactorial condition predominantly influenced by local anatomical and environmental factors, with genetic predisposition acting as a secondary contributor. Early diagnosis and appropriate imaging assessment are essential to prevent complications such as root resorption and to optimize treatment outcomes.

## 1. Introduction

Tooth eruption is a complex, clinically relevant biological process involving coordinated interactions among dental follicle signaling pathways, alveolar bone remodeling, and periodontal ligament activity. Disturbances in this highly regulated mechanism may lead to tooth impaction, defined as the failure of a tooth to erupt into its normal functional position within the dental arch during the expected developmental period [1]. Impaction is among the most frequently encountered developmental dental anomalies in clinical practice and may result in functional, esthetic, and orthodontic complications.

Among permanent teeth, third molars exhibit the highest rates of impaction; however, permanent canines are recognized as the second most commonly affected teeth [2,3]. Their susceptibility to eruption disturbances is largely attributed to specific anatomical and developmental characteristics, including a prolonged eruption pathway, delayed eruption timing, and a strategic position within the dental arch. Given their essential roles in occlusal guidance, arch stability, and facial aesthetics, canine impaction can significantly compromise functional outcomes and increase orthodontic treatment complexity [3].

Epidemiological studies have consistently demonstrated considerable variability in the prevalence of canine impaction across populations. Reported frequencies for impacted maxillary canines typically range from approximately 0.97% to 7.10%, whereas mandibular impaction occurs far less frequently, with estimates generally between 0.3% and 2.8% [4,5,6]. In addition, marked differences in positional distribution have been documented, with palatal displacement representing the most common anatomical presentation, followed by buccal and mid-alveolar locations [7,8].

A clear distinction must be made between impacted and retained teeth. An impacted tooth is characterized by scarce eruptive potential, usually presenting with complete root formation and absence of active periodontal ligament function. In contrast, a retained tooth remains embedded due to mechanical obstruction or abnormal positioning but may retain eruptive capability if the causative obstacle is eliminated [2]. Although impacted teeth typically remain near their developmental site, rare cases of transmigration may occur, particularly involving mandibular canines. Transmigration refers to the pre-eruptive migration of a tooth across the midline and represents an uncommon but clinically significant eruption disturbance associated with abnormal developmental pathways [5,9].

The etiology of canine impaction is widely recognized as multifactorial and incompletely understood. Current evidence suggests a complex interaction between local anatomical factors, environmental influences, skeletal characteristics, and genetic predisposition. Frequently reported local contributors include overretained deciduous canines, dental arch constriction, supernumerary teeth, odontomas, and mechanical obstruction within the eruption pathway [10,11]. Genetic associations have also been documented, particularly involving lateral incisor anomalies such as agenesis, microdontia, or morphological alterations, which may disrupt normal eruptive guidance mechanisms [12].

Advances in diagnostic imaging have significantly improved the evaluation and management of impacted canines. While panoramic radiography remains widely used for initial screening, its diagnostic accuracy is limited by two-dimensional projection and anatomical superimposition. In contrast, CBCT provides precise three-dimensional localization (3D), enhanced assessment of surrounding bone characteristics, and early detection of root resorption affecting adjacent teeth, thereby substantially improving diagnostic reliability and treatment planning [13,14,15].

Despite extensive research, considerable variability persists in reported epidemiological data, etiological interpretations, and diagnostic approaches across populations and study designs [6,16]. This heterogeneity underscores the need for a comprehensive synthesis of contemporary evidence to clarify the incidence patterns, anatomical characteristics, etiological determinants, and diagnostic considerations associated with permanent canine impaction.

Despite the growing body of literature, important gaps remain in integrating epidemiological, anatomical, and etiological findings into a unified clinical framework. Many previously published studies have focused on isolated aspects such as prevalence or imaging, without providing a comprehensive synthesis of interacting risk factors and their clinical implications. Furthermore, variability in diagnostic criteria and imaging modalities has limited comparability across studies. Addressing these gaps is essential for improving early diagnosis, risk stratification, and treatment planning.

Therefore, this systematic review critically evaluates the current literature on the prevalence, positional distribution, etiological factors, and imaging assessment of permanent canine impaction, drawing on contemporary epidemiological, radiographic, and clinical studies.

## 2. Materials and Methods

### 2.1. Study Design

**Figure 1:**
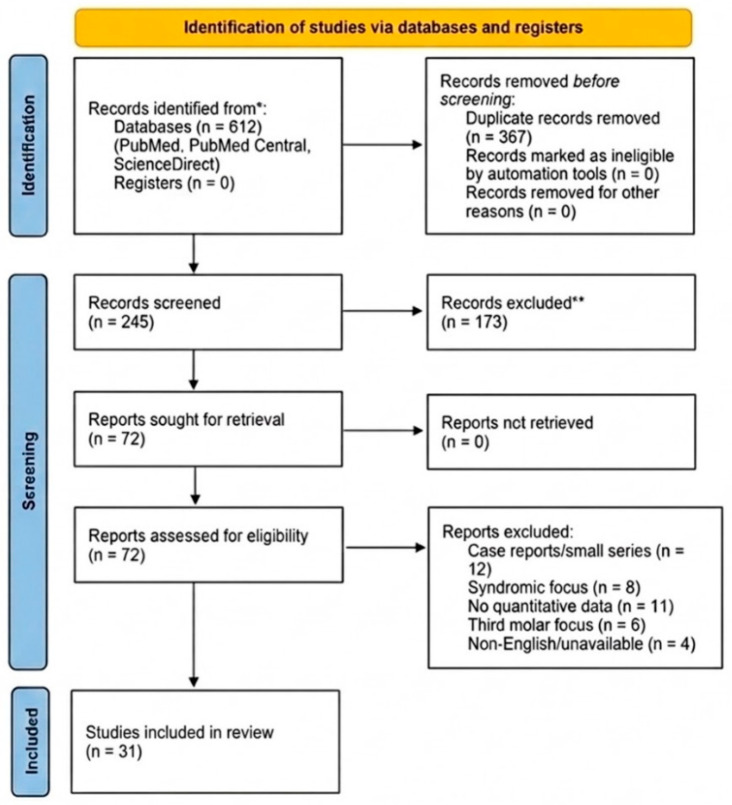
PRISMA 2020 flow diagram illustrating the process of study identification, screening, eligibility assessment, and inclusion. Records marked with “*” indicate studies removed before screening. Records marked with “**” indicate studies excluded during title and abstract screening.

This study was designed as a systematic review to synthesize available evidence regarding the prevalence, anatomical distribution, etiological determinants, and diagnostic assessment of permanent canine impaction. The review was conducted in accordance with the Preferred Reporting Items for Systematic Reviews and Meta-Analyses (PRISMA) 2020 guidelines. The methodology was predefined before data extraction to ensure transparency and reproducibility. The review protocol was not prospectively registered. A completed PRISMA 2020 checklist is provided in the Supplementary Materials [17], and the study selection process is illustrated using the PRISMA 2020 flow diagram (Figure 1). Ethical approval for the study framework was obtained from the Research Ethics Subcommittee of the University of Oradea (Approval No. 28, dated 30 June 2025; Registration No. 10312/30.06.2025). Following the screening and eligibility assessment, 31 studies met the predefined inclusion criteria and were included in the qualitative synthesis.

The review question was structured according to a PICO-inspired framework, where the population consisted of patients with permanent canine impaction, the condition of interest included prevalence, anatomical distribution, etiological factors, and imaging characteristics, and the outcomes involved epidemiological patterns and diagnostic implications. Due to heterogeneity of study designs and outcomes, a qualitative systematic synthesis approach was adopted.

### 2.2. Search Strategy

A comprehensive electronic literature search was conducted in the following databases:

- PubMed
- PubMed Central
- ScienceDirect

The final search was performed on 31 December 2025. Manual searches were additionally conducted in leading orthodontic and oral radiology journals to identify potentially eligible studies not captured through electronic databases.

Two independent reviewers screened titles and abstracts. Full texts of potentially eligible articles were assessed for inclusion. Discrepancies were resolved through discussion and consensus. Disagreements between reviewers primarily involved the eligibility of borderline studies, particularly those with mixed populations or limited etiological data. These disagreements were resolved through discussion and consensus. Both reviewers were clinicians with experience in orthodontics and dentomaxillofacial radiology, each with more than five years of clinical and academic experience. When consensus could not be reached, a third senior reviewer was consulted.

The search strategy employed combinations of Medical Subject Headings (MeSH) terms and free-text keywords related to canine impaction, prevalence, etiology, transmigration, and imaging. Boolean operators (AND, OR) and parentheses were used to structure database-specific search syntax [3,11].

The complete electronic search strategies for each database are provided below and in Supplementary Table S1. Inclusion and exclusion criteria were applied for study selection (Table 1).

### 2.3. Eligibility Criteria

#### 2.3.1. Inclusion Criteria

Studies were included if they:

- Investigated permanent canine impaction
- Reported prevalence, anatomical distribution, or etiological factors
- Used clinical or radiographic diagnostic methods
- Were peer-reviewed publications
- Were written in English

#### 2.3.2. Exclusion Criteria

Studies were excluded if they:

- Focused exclusively on syndromic or systemic conditions
- Consisted solely of case reports or small case series
- Lacked quantitative epidemiological data
- Investigated exclusively third molar impaction

### 2.4. Data Extraction and Synthesis

Data extracted from eligible studies included:

- Sample size
- Prevalence rates
- Anatomical positioning
- Gender distribution
- Etiological factors
- Imaging modalities
- Presence of root resorption

Due to methodological heterogeneity among studies, quantitative meta-analysis was not feasible. Therefore, a qualitative synthesis approach was adopted. However, to enhance analytical depth, ranges, frequency distributions, and comparative trends across studies were systematically evaluated and summarized.

### 2.5. Risk of Bias Assessment

The methodological quality of included studies was evaluated using the Joanna Briggs Institute (JBI) Critical Appraisal Tools appropriate for observational study designs [18]. Given that the majority of included investigations were cross-sectional or retrospective cohort studies, the JBI Critical Appraisal Checklist for Analytical Cross-Sectional Studies and the Checklist for Cohort Studies were applied as appropriate.

The JBI appraisal domains included:

- Clearly defined inclusion criteria
- Detailed description of study participants and setting
- Valid and reliable measurement of exposure and outcome variables
- Use of objective and standardized diagnostic criteria
- Identification and management of confounding factors
- Appropriate statistical analysis
- Adequacy of follow-up (for cohort designs)

Two reviewers independently evaluated each study. Discrepancies were resolved by consensus.

Studies were categorized as presenting:

- Low risk of bias (≥75% criteria satisfied)
- Moderate risk of bias (50–74% criteria satisfied)
- High risk of bias (<50% criteria satisfied)

Common methodological limitations included retrospective design, convenience-based sampling from orthodontic populations, limited reporting of examiner calibration, and insufficient control of potential confounding variables.

Risk-of-bias assessments were incorporated into the qualitative synthesis but were not used as exclusion criteria.

## 3. Results

Thirty-one studies met the inclusion criteria and were included in the qualitative synthesis. The characteristics of included studies are summarized in Table 2.

Proposed mechanisms are presented in Table 3.

### 3.1. Study Characteristics and Population Overview

The included studies demonstrated substantial heterogeneity in sample size, population characteristics, diagnostic criteria, and methodological design. Sample sizes ranged from small radiographic cohorts of fewer than 100 individuals to large epidemiological investigations involving over 6000 participants.

Across studies evaluating general dental anomalies, impacted teeth accounted for approximately 3.9–23.5% of the examined populations. In contrast, impacted canines accounted for a smaller proportion, ranging from 0.95% to 10.5%, depending on the study population and diagnostic method employed [8,16,18,19]. Higher prevalence values approaching 10% were observed in orthodontic or referral-based cohorts, whereas general population studies consistently reported lower ranges between 1% and 3% [3,6]. Studies of orthodontic populations have reported higher frequencies of canine impaction than general population studies, likely reflecting referral bias and increased detection through routine radiographic screening [19,20,21].

Overall, the epidemiological data confirm that canine impaction is relatively uncommon compared with other eruption disturbances, yet it remains clinically significant due to its potential complications and treatment complexity.

The study selection process followed the PRISMA 2020 flow diagram and is presented in Figure 1. A total of 612 records were initially identified through electronic database searches. After removal of duplicate entries, 245 records remained for title and abstract screening. Following this initial evaluation, 72 articles were assessed for full-text eligibility. Ultimately, 31 studies met the predefined inclusion criteria and were included in the qualitative synthesis. Detailed extracted quantitative data from the included studies are provided in the Supplementary Materials (Tables S2–S6). Detailed characteristics of epidemiological studies reporting prevalence data are summarized in Table 4.

Variability in reported prevalence may reflect differences in study design, imaging modality, and population type (general vs. orthodontic referral cohorts). A comparative assessment of prevalence ranges across study types revealed consistently higher values in orthodontic populations (up to approximately 10%) compared with general population studies (typically 1–3%), suggesting a strong influence of referral and selection bias.

### 3.2. Risk of Bias Across Studies

Methodological appraisal indicated that most included studies demonstrated a moderate risk of bias, primarily attributable to retrospective design and non-random sampling strategies. Reporting of examiner calibration and confounder control was inconsistent across studies. No investigation was excluded based on methodological quality; however, identified limitations were considered during qualitative synthesis.

### 3.3. Prevalence and Anatomical Distribution of Impacted Canines

Maxillary canine impaction was consistently reported to occur more frequently than mandibular impaction. Reported prevalence ranged from 0.97% to 7.10% for maxillary canines and from 0.3% to 2.8% for mandibular canines [4,5,6,16]. This marked discrepancy reflects differences in eruption pathways, anatomical constraints, and developmental timing between maxillary and mandibular dentition.

Regarding positional distribution, palatal impaction represented the most common anatomical pattern. Across studies, palatal displacement accounted for approximately 25% to 74% of impacted maxillary canines [7,8,22]. Buccal or labial impactions demonstrated lower frequencies, generally ranging between 13% and 38%, whereas mid-alveolar positioning showed intermediate prevalence in several populations [7,8].

Significant variability in positional patterns was observed among populations. Certain studies reported predominance of palatal impaction, whereas others identified higher frequencies of labial or mid-alveolar displacement. These variations may reflect ethnic differences, genetic influences, environmental factors, and diagnostic methodologies [22].

Mandibular canine impaction exhibited distinct characteristics compared with maxillary impaction. It was most commonly located buccally and demonstrated a higher tendency for migration and transmigration phenomena [5,9].

### 3.4. Age and Gender Distribution

The majority of studies reported a higher prevalence of canine impaction among females compared with males, with female-to-male ratios ranging from approximately 1.3:1 to 2:1 [6,16,23]. This gender predilection has been attributed to earlier eruption timing in females, differences in craniofacial growth patterns, and potential genetic influences [3].

However, some investigations failed to identify statistically significant gender differences, particularly in mandibular canine impaction and transmigration cases [5,9]. These findings suggest that gender-related influences may vary with population characteristics and anatomical location [24].

The mean age of affected individuals across studies ranged from 14 to 26 years, reflecting the typical age at clinical detection during orthodontic evaluation [16,23]. Early diagnosis is particularly important because the likelihood of spontaneous eruption decreases significantly after completion of root development [25].

### 3.5. Root Resorption Associated with Impacted Canines

Root resorption of adjacent teeth represents one of the most clinically significant complications associated with canine impaction. Multiple studies consistently demonstrated that lateral incisors are the most frequently affected teeth due to their anatomical proximity to the eruptive path of maxillary canines [26,27].

Reported rates of lateral incisor resorption ranged from approximately 25% to 80% depending on imaging modality and diagnostic criteria [24,25,26,27]. Central incisors exhibited lower resorption rates, generally between 5% and 32%, while first premolars demonstrated the lowest susceptibility [26,28].

These findings support the widely accepted theory that anatomical proximity and physical pressure exerted by ectopically positioned canines play a major role in the pathogenesis of root resorption [26].

Importantly, studies using CBCT reported substantially higher detection rates than conventional radiography, highlighting the superior sensitivity of 3D imaging techniques [13,14].

### 3.6. Transmigration of Canines

Transmigration represents a rare but clinically significant phenomenon characterized by the migration of an unerupted canine across the midline. Reported canine transmigration prevalence rates ranged between approximately 0.10% and 5.4%, with mandibular canines demonstrating significantly higher susceptibility than maxillary canines [5,9,29].

Unilateral transmigration was more frequently observed than bilateral cases. Among the different patterns described by Mupparapu’s classification, Type I transmigration was reported as the most common, accounting for approximately 64.5% of cases [30].

Despite its rarity, transmigration presents diagnostic and therapeutic challenges due to unpredictable eruption patterns and potential damage to adjacent anatomical structures.

### 3.7. Local Etiological Factors

Local environmental factors represent the most consistently identified etiological contributors to canine impaction [31]. Overretained deciduous canines have been repeatedly reported as a major risk factor, particularly when root resorption fails to occur normally [10,11]. Clinical observations indicate that the extraction of retained primary canines may facilitate the spontaneous eruption of displaced permanent canines in certain cases [10].

Other local factors include supernumerary teeth, odontomas, cystic lesions, and trauma, all of which may mechanically obstruct the eruption pathway [11,32]. Narrow maxillary arch dimensions and dental crowding have also been associated with an increased risk of impaction [33].

Chronic periapical pathology related to retained deciduous teeth may further contribute to eruption disturbances by creating physical barriers within the alveolar bone [34,35].

### 3.8. Genetic and Developmental Factors

Genetic influences have been implicated in canine impaction, as evidenced by associations with other dental anomalies. Alterations in lateral incisor morphology, including agenesis, microdontia, and peg-shaped crowns, are frequently observed in patients with impacted canines [12,36]. In addition to morphological anomalies, shortened lateral incisor roots have also been suggested as a potential contributing factor. Reduced root length may alter the eruptive guidance pathway, thereby increasing the risk of palatal displacement of maxillary canines [24].

Although bilateral expression would be expected for purely genetic conditions, epidemiological studies indicate that most canine impactions are unilateral, suggesting that environmental and local factors play a dominant etiological role [12].

Genetic factors are therefore considered contributory rather than primary determinants of impaction.

### 3.9. Imaging Modalities in Diagnosis

Across included studies, panoramic radiography was performed using standard digital orthopantomography units, while CBCT imaging was conducted using various devices with voxel resolutions typically ranging between 0.125 mm and 0.4 mm, depending on study protocols. Panoramic radiography remains widely used for initial screening; however, its predictive accuracy for determining canine position and assessing root resorption is limited [13].

CBCT has demonstrated superior diagnostic performance due to its ability to provide 3D visualization, eliminate anatomical superimposition, and accurately assess spatial relationships [14,15]. CBCT also enables evaluation of bone density, trabecular structure, and volumetric characteristics surrounding impacted teeth [37].

These advantages make CBCT the preferred imaging modality for complex cases, surgical planning, and early detection of complications. Table 5 illustrates the advantages and limitations of imaging diagnosis modalities.

## 4. Discussion

### 4.1. Overview of Key Findings

This systematic review synthesized contemporary evidence regarding the epidemiology, anatomical distribution, etiological determinants, and diagnostic evaluation of permanent canine impaction. The findings confirm that canine impaction is a relatively common developmental dental anomaly with a multifactorial etiology involving local anatomical factors, skeletal characteristics, and genetic predisposition.

Consistent with previous epidemiological reports, maxillary canines were found to be significantly more frequently impacted than mandibular canines, with prevalence estimates ranging from approximately 1% to 7% depending on the studied population [3,4,5,6]. This discrepancy is largely explained by differences in eruption pathways, spatial constraints within the maxilla, and the prolonged developmental period of maxillary canines [3].

The review also confirms that palatal displacement represents the predominant positional pattern, supporting long-standing observations in orthodontic literature [7,22]. However, considerable variability among populations suggests that both environmental and genetic influences contribute to positional differences.

Importantly, the integration of findings across epidemiological, anatomical, and etiological domains highlights the necessity of a multidisciplinary diagnostic approach. The variability observed across populations further suggests that canine impaction cannot be explained by a single causative model but rather by dynamic interactions between genetic susceptibility and environmental constraints [5,7,22,23].

### 4.2. Multifactorial Etiology of Canine Impaction

#### 4.2.1. Dominant Role of Local Environmental Factors

The strongest and most consistent evidence identified in this review supports the primary role of local environmental factors in the development of canine impaction [31,32]. Overretained deciduous canines were repeatedly reported as one of the most significant contributors, particularly when root resorption fails to occur physiologically [10,11]. This finding aligns with the well-established clinical observation that early extraction of retained primary canines may promote spontaneous eruption of displaced permanent canines.

Mechanical obstruction by supernumerary teeth, odontomas, cystic lesions, or trauma has also been widely documented as a direct etiological mechanism [11,31,32]. These factors interfere with normal eruption pathways, resulting in altered tooth angulation or displacement.

Additionally, maxillary transverse deficiency and dental arch constriction were frequently associated with palatal impaction, suggesting that spatial limitations play a crucial role in eruption failure [33]. This relationship supports the concept that environmental constraints within the alveolar bone environment may be more influential than intrinsic developmental abnormalities [25].

Overall, the accumulated evidence indicates that local anatomical and mechanical factors represent the most significant determinants of canine impaction.

#### 4.2.2. Skeletal and Morphological Influences

Advances in three-dimensional imaging have revealed important skeletal characteristics associated with canine impaction. Studies utilizing CBCT demonstrated increased bone density, altered trabecular microstructure, and volumetric differences in the maxilla surrounding impacted canines [37,38].

These findings suggest that eruption disturbances may be influenced not only by dental positioning but also by the surrounding osseous environment. Increased bone density may impede eruptive movement, while variations in trabecular architecture could alter eruption pathways [37,38,39].

However, the causal relationship between skeletal characteristics and impaction remains unclear. These bone alterations may represent secondary adaptive responses rather than primary etiological factors [25,40].

#### 4.2.3. Genetic Contributions and Developmental Associations

Genetic influences in canine impaction have been supported by consistent associations with other dental anomalies, particularly lateral incisor agenesis, microdontia, and peg-shaped morphology [12,36,41]. These findings support the “guidance theory,” which proposes that abnormal lateral incisor development disrupts the eruptive guidance pathway of the canine [24,42,43].

Nevertheless, epidemiological evidence indicates that most canine impactions occur unilaterally, which argues against a purely genetic etiology [12,43,44,45]. Instead, genetic factors likely act as predisposing influences that increase susceptibility when combined with environmental or anatomical disturbances [45,46].

Thus, canine impaction should be considered a condition resulting from complex interactions between genetic predisposition and local environmental factors rather than a strictly hereditary disorder [23,39,45]. This multifactorial model underscores the importance of early risk assessment protocols that combine clinical, radiographic, and developmental indicators to identify patients at increased risk of impaction [25,42,44].

### 4.3. Clinical Significance of Root Resorption

One of the most clinically important findings highlighted in this review is the high prevalence of root resorption affecting adjacent teeth, particularly lateral incisors [26,27,28,44,45]. This complication occurs due to the close anatomical proximity between ectopically positioned canines and neighboring roots.

The reported incidence of lateral incisor resorption varied widely, ranging from approximately 25% to 80% depending on diagnostic methodology. Studies using CBCT reported higher detection rates than conventional radiography, underscoring the limitations of two-dimensional imaging [13,25,42,44].

Early detection of impacted canines is therefore essential to prevent irreversible damage to adjacent teeth and to improve orthodontic treatment outcomes.

### 4.4. Transmigration: A Rare but Clinically Relevant Phenomenon

Although rare, canine transmigration represents a unique eruption disturbance with significant diagnostic and therapeutic implications [47,48]. Mandibular canines demonstrated a greater tendency for transmigration compared with maxillary canines, likely due to anatomical differences in bone density and eruption pathways [5,9,48].

The predominance of unilateral cases and the distribution patterns described in Mupparapu’s classification suggest that transmigration may be influenced by abnormal eruptive forces rather than solely by genetic predisposition [30].

Given its unpredictable progression, early radiographic detection is critical for appropriate management planning.

### 4.5. Diagnostic Imaging and Clinical Decision-Making

The findings of this review strongly support the superior diagnostic value of CBCT compared with panoramic radiography in assessing canine impaction. CBCT provides precise 3D localization, accurate evaluation of root resorption, and detailed assessment of surrounding bone structures [13,24,42,44].

However, routine use of CBCT must be carefully balanced against radiation exposure considerations, particularly in pediatric populations. Current evidence suggests that CBCT should be reserved for cases requiring detailed spatial assessment, complex surgical planning, or evaluation of suspected complications [15,49,50].

Thus, imaging protocols should be guided by clinical findings and individualized risk-benefit assessment.

### 4.6. Clinical Implications

The clinical implications of these findings are substantial (Table 6). Early diagnosis during mixed dentition is critical for implementing interceptive treatment strategies, such as extracting retained primary canines or managing orthodontic space. Interceptive approaches include timely extraction of retained primary canines between the ages of 10 and 13 years, space management within the dental arch, and monitoring of eruptive position through periodic radiographic evaluation [50,51].

Additionally, recognition of associated risk factors, including lateral incisor anomalies, arch constriction, and delayed eruption patterns, can improve early identification of high-risk patients. Early screening using panoramic radiography is recommended between 9 and 11 years of age, when canine displacement can be identified before completion of root development [42,43,44,50,51,52].

Interdisciplinary collaboration between orthodontists, oral surgeons, and radiologists is essential for optimal management of impacted canines [22,25,42,44,50,52].

### 4.7. Limitations of the Evidence

Several limitations must be considered when interpreting the findings of this review. First, substantial heterogeneity exists among included studies regarding diagnostic criteria, imaging methods, and population characteristics [35,53]. This variability limited the feasibility of quantitative meta-analysis.

Second, many studies were retrospective in design, introducing potential selection bias and limiting causal inference. Differences in imaging modalities may also have influenced reported prevalence rates, particularly in the detection of root resorption. The absence of standardized diagnostic criteria across studies represents an additional limitation affecting comparability.

Increasing use of CBCT in recent years may have artificially elevated reported detection rates of root resorption and certain positional patterns.

Finally, geographic and ethnic diversity among study populations may affect the generalizability of findings [35,53].

Future multicenter studies employing standardized CBCT-based diagnostic protocols are necessary to refine prevalence estimates and elucidate causal mechanisms.

### 4.8. Strengths and Novel Contributions of the Review

This systematic review provides several important contributions to the current body of knowledge. First, it integrates epidemiological, anatomical, etiological, and radiographic evidence into a single comprehensive framework, allowing for a more holistic understanding of permanent canine impaction.

Second, it highlights the relative contribution of local versus genetic factors, clarifying ongoing debates regarding etiological predominance.

Third, the review emphasizes the diagnostic superiority of CBCT and its implications for early detection and treatment planning.

Finally, by synthesizing heterogeneous data across multiple populations, this study identifies consistent patterns while also acknowledging variability, thereby supporting both generalized and population-specific clinical approaches.

## 5. Conclusions

Permanent canine impaction represents a multifactorial developmental dental anomaly with significant clinical consequences. The evidence synthesized in this review demonstrates that maxillary canines are substantially more frequently impacted than mandibular canines, with palatal displacement being the predominant positional pattern.

Local environmental factors, particularly overretained deciduous canines, mechanical obstruction, and dental arch constriction, appear to play the most influential etiological role. Skeletal characteristics and genetic predisposition contribute as secondary modifying factors rather than primary causes.

Root resorption of adjacent teeth, especially lateral incisors, represents the most significant complication and underscores the importance of early detection. CBCT provides superior diagnostic accuracy and is essential for comprehensive evaluation in complex cases.

Early identification and timely interceptive treatment remain critical for preventing complications, minimizing treatment complexity, and improving long-term outcomes.

The present review contributes to the existing literature by providing an integrated synthesis of current evidence and identifying key gaps that warrant further investigation. These findings support the development of improved diagnostic protocols and targeted preventive strategies, ultimately enhancing patient outcomes in clinical orthodontic practice.

## Figures and Tables

**Table 1:** Inclusion and exclusion criteria applied for study selection.

Inclusion Criteria	Exclusion Criteria
Studies investigating permanent canine impaction	Case reports and small case series
Epidemiological, clinical, or radiographic studies	Studies focusing on syndromic conditions
Human subject studies	Non-English publications
Studies providing quantitative data	Studies limited to third molar impaction
Prospective, retrospective, and cross-sectional observational studies	Case reports, narrative reviews, and expert opinions

**Table 2:** Summary characteristics of the included studies (n = 31).

Study Category	Number of Studies	Sample Size Range	Principal Findings
**Epidemiological prevalence studies**	11	200–6000 participants	Prevalence ranged from 0.97% to 7.1%
**Radiographic positional analyses**	8	50–1200 patients	Palatal displacement was predominant
**Root resorption investigations**	5	80–500 patients	Lateral incisors most frequently affected
**Transmigration studies**	4	30–400 cases	Mandibular canines more commonly involved
**Etiological factor analyses**	3	100–800 subjects	Local mechanical factors most influential

**Table 3:** Principal etiological mechanisms associated with permanent canine impaction.

Mechanism	Description	Clinical Implication
**Local mechanical obstruction**	Retained deciduous teeth, supernumerary teeth	Eruption pathway interference
**Arch space deficiency**	Narrow maxillary arch	Increased impaction risk
**Genetic predisposition**	Lateral incisor anomalies	Altered eruption guidance
**Skeletal bone density variation**	Increased trabecular density	Impaired eruptive movement

**Table 4:** Characteristics of Epidemiological Studies Reporting Prevalence of Permanent Canine Impaction.

Study (First Author, Year)	Country	Study Design	Sample Size	Prevalence of Canine Impaction (%)	Imaging Method	Population Type
**Aljehani, 2023 [20]**	Saudi Arabia	Cross-sectional	2000	3.1	Panoramic radiography	General
**Bilge et al., 2018 [16]**	Turkey	Retrospective	2350	2.9	Digital panoramic	General
**Di Spirito et al., 2022 [19]**	Italy	Cross-sectional	600	5.2	Panoramic radiography	Orthodontic
**Sella Tunis et al., 2021 [21]**	Israel	Retrospective	1200	4.8	Panoramic radiography	Orthodontic
**Yang et al., 2022 [22]**	South Korea	Retrospective	850	2.4	CBCT	Orthodontic
**Viktoraviciute et al., 2023 [23]**	Lithuania	Retrospective	500	3.7	CBCT	Orthodontic
**Nemec et al., 2024 [4]**	Austria	Retrospective	1100	1.9	CBCT	General
Aydin et al., 2004 * [6]	Turkey	Cross-sectional	4500	2.3	Panoramic radiography	General

* Included due to relevance for transmigration epidemiology.

**Table 5:** Diagnostic imaging modalities for evaluation of impacted canines.

Imaging Modality	Advantages	Limitations
Panoramic radiography	Initial screening tool	Two-dimensional limitations
Periapical radiographs	Local detail assessment	Limited spatial information
CBCT	Accurate 3D localization and resorption detection	Higher radiation dose

**Table 6:** Clinical management approaches for impacted canines.

Treatment Strategy	Indications	Outcomes
Interceptive extraction of primary canine	Early displacement	Facilitates spontaneous eruption
Orthodontic traction	Moderate impaction	High success rate
Surgical exposure	Deep impaction	Enables orthodontic alignment
Surgical removal	Severe displacement	Prevents complications

## Data Availability

No new data were created or analyzed in this study. Data sharing is not applicable to this article.

## Supplementary Materials

The following supporting information can be downloaded at: https://www.mdpi.com/article/10.3390/medicina62040681/s1, PRISMA 2020 checklist and detailed search strategies [17]. The PRISMA 2020 flow diagram template used to generate Figure 1 is also provided.

## References

[1] Guarnieri R., Germanò F., Sottile G., Barbato E., Cassetta M. (2024). Local factors relating to mandibular canine impaction: A retrospective study. Am. J. Orthod. Dentofac. Orthop..

[2] Laganà G., Venza N., Borzabadi-Farahani A., Fabi F., Danesi C., Cozza P. (2017). Dental anomalies: Prevalence and associations between them in a large sample of non-orthodontic subjects—A cross-sectional study. BMC Oral Health.

[3] Bedoya M.M., Park J.H. (2009). A review of the diagnosis and management of impacted maxillary canines. J. Am. Dent. Assoc..

[4] Nemec M., Garzarolli-Thurnlackh G., Lettner S., Nemec-Neuner H., Gahleitner A., Stavropoulos A., Bertl K., Jonke E. (2024). Prevalence and characteristics of and risk factors for impacted teeth with ankylosis and replacement resorption—A retrospective, 3D-radiographic assessment. Prog. Orthod..

[5] Koç A., Kaya S., Abdulsalam W.A. (2021). Three-dimensional analysis of impacted maxillary and mandibular canines and evaluation of factors associated with transmigration on cone-beam computed tomography images. J. Oral Maxillofac. Surg..

[6] Aydin U., Yilmaz H.H., Yildirim D. (2004). Incidence of canine impaction and transmigration in a patient population. Dentomaxillofac. Radiol..

[7] Eid F.Y., Ghaleb S.I., Badr F.F., Marzouk E.S. (2024). Three-dimensional assessment of the skeletal characteristics accompanying unilateral maxillary canine impaction: A retrospective cone-beam computed tomography study. BMC Oral Health.

[8] MacDonald D., Yu W. (2020). Incidental findings in a consecutive series of digital panoramic radiographs. Imaging Sci. Dent..

[9] Celikoglu M., Kamak H., Oktay H. (2010). Investigation of transmigrated and impacted maxillary and mandibular canine teeth in an orthodontic patient population. J. Oral Maxillofac. Surg..

[10] Becker A., Chaushu S. (2015). Etiology of maxillary canine impaction: A review. Am. J. Orthod. Dentofac. Orthop..

[11] Sajnani A.K. (2015). Permanent maxillary canines—Review of eruption pattern and local etiological factors leading to impaction. J. Investig. Clin. Dent..

[12] Brook A.H., Jernvall J., Smith R.N., Hughes T.E., Townsend G.C. (2014). The dentition: The outcomes of morphogenesis leading to variations of tooth number, size and shape. Aust. Dent. J..

[13] Peralta-Mamani M., Rubira C.M., López-López J., Honório H.M., Rubira-Bullen I.R. (2024). CBCT vs. panoramic radiography in assessment of impacted upper canine and root resorption of the adjacent teeth: A systematic review and meta-analysis. J. Clin. Exp. Dent..

[14] Alqerban A., Jacobs R., Fieuws S., Willems G. (2011). Comparison of two cone beam computed tomographic systems versus panoramic imaging for localization of impacted maxillary canines and detection of root resorption. Eur. J. Orthod..

[15] Botticelli S., Verna C., Cattaneo P.M., Heidmann J., Melsen B. (2011). Two- versus three-dimensional imaging in subjects with unerupted maxillary canines. Eur. J. Orthod..

[16] Bilge N.H., Yeşiltepe S., Törenek Ağırman K., Çağlayan F., Bilge O.M. (2018). Investigation of prevalence of dental anomalies by using digital panoramic radiographs. Folia Morphol..

[17] Page M.J., McKenzie J.E., Bossuyt P.M., Boutron I., Hoffmann T.C., Mulrow C.D., Shamseer L., Tetzlaff J.M., Akl E.A., Brennan S.E. (2021). The PRISMA 2020 statement: An updated guideline for reporting systematic reviews. BMJ.

[18] Hilton M. (2024). JBI Critical appraisal checklist for systematic reviews and research syntheses. J. Can. Health Libr. Assoc..

[19] Di Spirito F., Scelza G., Amato A., Rosa D., Gallotti A., Martina S. (2022). Prevalence of dental anomalies in a sample of growing subjects: A retrospective study. Epidemiol. Prev..

[20] Aljehani D.K. (2023). Prevalence of canine impaction in the western province of Saudi Arabia: A cross-sectional survey. J. Orthod. Sci..

[21] Tunis S., Sarne O., Hershkovitz I., Finkelstein T., Pavlidi A.M., Shapira Y., Davidovitch M., Shpack N. (2021). Dental anomalies’ characteristics. Diagnostics.

[22] Yang J.S., Cha J.Y., Lee J.Y., Choi S.H. (2022). Radiographical characteristics and traction duration of impacted maxillary canine requiring surgical exposure and orthodontic traction: A cross-sectional study. Sci. Rep..

[23] Viktoraviciute V., Mockute G., Smailiene D. (2023). Comparative analysis of morphological characteristics and localization of maxillary impacted canines using cone beam computed tomography. Med. Sci. Monit..

[24] Fernández-Polo P., Aguayo-Linares G., Martínez-Madero E., Montarelo J., Pérez-Martín T., Martin C. (2025). Three-Dimensional Analysis of the Association Between the Characteristics of the Included Maxillary Canines and the Lateral Incisors. Appl. Sci..

[25] Dipalma G., Inchingolo A.M., Morolla R., Inchingolo F., Venere D.D., Maspero C., Palermo A., Marinelli G., Inchingolo A.D. (2026). Association Between Bone Density and Maxillary Canine Impaction: A CBCT-Based Study. J. Clin. Med..

[26] Simić S., Nikolić P., Stanišić Zindović J., Jovanović R., Stošović Kalezić I., Djordjević A., Popov V. (2022). Root resorptions on adjacent teeth associated with impacted maxillary canines. Diagnostics.

[27] Lai C.S., Bornstein M.M., Mock L., Heuberger B.M., Dietrich T., Katsaros C. (2013). Impacted maxillary canines and root resorptions of neighbouring teeth: A radiographic analysis using cone-beam computed tomography. Eur. J. Orthod..

[28] Aktı A., Dolunay U., Kaya D.I., Gürses G., Yeşil D. (2024). Evaluation of the relationship between impacted maxillary canine teeth and root resorption in adjacent teeth: A cross-sectional cone beam computed tomography study. Diagnostics.

[29] Aktan A.M., Kara S., Akgünlü F., Malkoç S. (2010). The incidence of canine transmigration and tooth impaction in a Turkish subpopulation. Eur. J. Orthod..

[30] Mupparapu M. (2002). Patterns of intra-osseous transmigration and ectopic eruption of mandibular canines: Review of literature and report of nine additional cases. Dentomaxillofac. Radiol..

[31] Golez A., Vrcon C., Ovsenik M. (2024). Jaw Morphology and Factors Associated with Upper Impacted Canines: Case-Controlled Trial. Appl. Sci..

[32] Shen Z., Wei J., Zhang J., Zhang Y., Yao J. (2025). The prevalence of dental agenesis, supernumerary teeth and odontoma in a Chinese paediatric population: An epidemiological study. BMC Oral Health.

[33] Hong W.H., Radfar R., Chung C.H. (2015). Relationship between the maxillary transverse dimension and palatally displaced canines: A cone-beam computed tomographic study. Angle Orthod..

[34] Cacciatore G., Poletti L., Sforza C. (2018). Early diagnosed impacted maxillary canines and the morphology of the maxilla: A three-dimensional study. Prog. Orthod..

[35] Agastra E., Saettone M., Parrini S., Cugliari G., Deregibus A., Castroflorio T. (2023). Impacted Permanent Mandibular Canines: Epidemiological Evaluation. J. Clin. Med..

[36] Yan B., Sun Z., Fields H., Wang L., Luo L. (2013). Etiologic factors for buccal and palatal maxillary canine impaction: A perspective based on cone-beam computed tomography analyses. Am. J. Orthod. Dentofac. Orthop..

[37] Köseoğlu Seçgin C., Karslıoğlu H., Özemre M.Ö., Orhan K. (2021). Gray value measurement for the evaluation of local alveolar bone density around impacted maxillary canine teeth using cone beam computed tomography. Med. Oral Patol. Oral Cir. Bucal.

[38] Sunal Akturk E., Toktas A.I., Can E., Kosen E., Sarica I. (2024). Assessment of the Trabecular Bone Microstructure Surrounding Impacted Maxillary Canines Using Fractal Analysis on Cone-Beam Computed Tomography Images. Diagnostics.

[39] Papadopoulou C.I., Sifakakis I., Tournis S. (2024). Metabolic Bone Diseases Affecting Tooth Eruption: A Narrative Review. Children.

[40] Manor Y., Kaganovich M., Gamliel M., Sadan N., Shmuly T. (2026). Factors Contributing to Complications and Failures of Impacted Canines Undergoing Surgical Orthodontic Treatment: A Retrospective Cohort Study. J. Clin. Med..

[41] Trybek G., Jaroń A., Gabrysz-Trybek E., Rutkowska M., Markowska A., Chmielowiec K., Chmielowiec J., Grzywacz A. (2023). Genetic Factors of Teeth Impaction: Polymorphic and Haplotype Variants of *PAX9*, *MSX1*, *AXIN2*, and *IRF6* Genes. Int. J. Mol. Sci..

[42] Hočevar M., Ovsenik M., Golež A. (2025). Position of Maxillary Lateral Incisor and First Premolar in Impaction of Maxillary Canines: A Controlled Clinical CBCT and 3D Study Model Analysis. Dent. J..

[43] Ceraulo S., Barbarisi A., Oliva B., Moretti S., Caccianiga G., Lauritano D., Biagi R. (2025). Treatment Options in Impacted Maxillary Canines: A Literature Review. Dent. J..

[44] Kucukkaraca E. (2023). Characteristics of Unilaterally Impacted Maxillary Canines and Effect on Environmental Tissues: A CBCT Study. Children.

[45] Iacob A.M., Escobedo Martínez M.F., Olay García S., Junquera Olay S., Junquera Gutiérrez L.M. (2024). Two-Dimensional Radiographic Diagnosis of Maxillary Canine Impactions. Dent. J..

[46] Pasini M., Giuca M.R., Ligori S., Mummolo S., Fiasca F., Marzo G., Quinzi V. (2020). Association between Anatomical Variations and Maxillary Canine Impaction: A Retrospective Study in Orthodontics. Appl. Sci..

[47] Zogakis I.P., Anagnostou C., Ioannidou I., Chaushu S., Papadopoulos M.A. (2025). Radiographic Evaluation of Impacted and Transmigrant Canines: Prevalence and Sex-Based Differences in an Orthodontic Cohort. Dent. J..

[48] Kuc A.E., Kotuła J., Kulgawczyk M., Kotuła K., Grzech-Leśniak Z., Zalewska A., Kulikowska-Kulesza J., Kawala B., Lis J., Sarul M. (2025). Orthodontic Treatment of a Transmigrating Impacted Lower Canine Using a Digitally Designed and 3D-Printed Lingual Appliance Combined with Corticotomy and Laser Therapy—A Case Report. J. Clin. Med..

[49] Păcurar C., Mesaroș O., Ștețiu A.A., Bucur S.M., Mihai C.N., Păcurar M. (2025). Impaction Predictors and Diagnostic Performance of CBCT Versus Panoramic Radiography for Supernumerary Teeth in a Romanian Multicenter Cohort. Diagnostics.

[50] Diaconu O.A., Gheorghiță L.M., Gheorghe A.G., Țuculină M.J., Munteanu M.C., Iacov C.A., Rădulescu V.M., Ionescu M., Mirea A.A., Bănică C.A. (2025). A Retrospective Study of CBCT-Based Detection of Endodontic Failures and Periapical Lesions in a Romanian Cohort. J. Clin. Med..

[51] Türker N., Yıldırım E.A., Bulut D.G., Ustaoğlu G. (2025). Effect of maxillary impacted canine teeth on root resorption of adjacent teeth: A CBCT-based observational study. BMC Oral Health.

[52] Fekonja A. (2024). Comparisons of Two Different Treatment Methods for Impacted Maxillary Canines: A Retrospective Study. J. Clin. Med..

[53] Martínez-González A., Montes-Díaz M.E., Gallardo-López N.E., Colino-Gallardo P., Criado-Pérez L., Alvarado-Lorenzo A. (2025). Prevalence of Palatally Displaced Canines and Their Association with Dental and Skeletal Anomalies: A Retrospective Study. Appl. Sci..

