# Supplementary material for: Permanent Canine Impaction: A Systematic Review of Incidence, Distribution, and Etiology
Source: Medicina (Kaunas). 2026 Apr 2;62(4):681. doi: 10.3390/medicina62040681 (PMC13118162; doi:10.3390/medicina62040681)
Supplement: Supplementary file 1 [file medicina-62-00681-s001.zip › medicina-4210328-supplementary.pdf]

SUPPLEMENTARY FILES

for: Incidence, Distribution Patterns, and Etiological Determinants of Permanent Canine Impaction: A Systematic Review of Epidemiological, Anatomical, Genetic, and Radiographic Evidence

Table S1. Full Electronic Search Strategies

PubMed

("canine impaction"[Title/Abstract] OR "impacted canine"[Title/Abstract]
OR "impacted maxillary canine"[Title/Abstract]
OR "mandibular canine impaction"[Title/Abstract])
AND
("prevalence"[Title/Abstract] OR "incidence"[Title/Abstract]
OR "epidemiology"[Title/Abstract]
OR "etiology"[Title/Abstract]
OR "transmigration"[Title/Abstract]
OR "cone-beam computed tomography"[Title/Abstract]
OR "CBCT"[Title/Abstract])
AND
("2009/12/01"[Date - Publication] : "2024/02/01"[Date - Publication])

PubMed Central

("maxillary canine impaction" OR "mandibular canine impaction"
OR "canine transmigration")
AND
("prevalence" OR "etiology" OR "root resorption"
OR "cone-beam computed tomography")
AND
(2009:2024[Publication Date])

Science Direct

TITLE-ABSTR-KEY("canine impaction" OR "impacted canine")
AND
TITLE-ABSTR-KEY("prevalence" OR "etiology" OR "transmigration"
OR "cone-beam computed tomography")
AND
PUBYEAR > 2009 AND PUBYEAR < 2025

Table S2. Prevalence of dental anomalies, impacted teeth, and impacted canines across included studies.

Study	Total Patients	Dental Anomalies (%)	Impacted Teeth (%)	Impacted Canines (%)
Study 6	6252	29	23.5	0.95
Study 7	4706	20.9	3.9	—
Study 8	4373	25.7	12	9.2
Study 9	1200	39.2	17.83	—
Study 10	400	—	—	4
Study 11	2897	36	14.9	10.5
Study 12	4500	—	—	3.58

Table S3. Positional distribution of impacted maxillary canines.

Study	Total Patients	Palatal (%)	Labial (%)	Mid-alveolar (%)
Study 12	64	67	28	5
Study 13	170	40.59	59.41	—
Study 14	40	65	22.5	12.5
Study 15	22	36.36	36.36	27.27
Study 2	4142	33.96	13.2	52.83
Study 16	1000	52.9	15.5	31.5
Study 17	89	74.1	23.1	2.8
Study 18	74	25.3	74.7	—
Study 19	134	58.6	15.4	25.9
Study 20	34	61.7	38.3	—
Study 21	113	51.49	30.6	17.91

Table S4. Age and gender distribution of patients with impacted canines.

Study	Total Patients	Mean Age	Females (%)	Males (%)
Study 6	6252	—	10.8	12.7
Study 22	102	16.25 y	57.84	42.16
Study 12	64	16.40 y	57.81	42.19
Study 23	151	24.94 y	66.9	33.1
Study 13	170	14.5 y	—	—
Study 1	102	14.8 y	—	—
Study 15	22	15.8 y	54.54	45.46
Study 2	4142	31 y	57	43
Study 17	89	18.3 y	62.5	37.5
Study 10	400	—	41.75	58.25
Study 19	134	26.7 y	67.9	31.3
Study 20	34	16.5 y	64.7	35.3

Table S5. Frequency of root resorption associated with impacted canines.

Study	Total Patients	Central Incisor (%)	Lateral Incisor (%)	First Premolar (%)
Study 13	170	18	27	10
Study 24	32	54.54	45.46	—
Study 19	134	32.1	58	19.1
Study 21	113	5.22	25.37	4.48

Table S6. Prevalence and distribution of canine transmigration.

Study	Total Patients	Unilateral (%)	Maxillary (%)	Mandibular (%)
Study 3	35	1.14	0.62	0.52
Study 20	4500	—	0.13	0.18
Study 25	2215	5.4	5.1	0.3
Study 26	24	—	0.34	0.14

PRISMA 2020 Checklist

Systematic Review Title:
Incidence, Distribution Patterns, and Etiological Determinants of Permanent Canine Impaction: A Systematic Review of Epidemiological, Anatomical, Genetic, and Radiographic Evidence

PRISMA 2020 Item	Section in Manuscript	Description of Compliance	Status
Title	Title Page	Identified explicitly as a systematic review	✅
Abstract	Abstract	Structured summary including background, objectives, methods, results, and conclusions	✅
Rationale	Introduction	Justified need for synthesis due to variability in epidemiological and diagnostic findings	✅
Objectives	Introduction (final paragraph)	Clearly stated aim to evaluate prevalence, positional distribution, etiological determinants, and imaging approaches	✅
Eligibility Criteria	Methods 2.3	Inclusion and exclusion criteria explicitly defined; summarized in Table 2	✅
Information Sources	Methods 2.2	Databases listed (PubMed, PubMed Central, ScienceDirect, orthodontic journals) with date range specified	✅
Search Strategy	Table 1	Full Boolean search strategy reported	✅
Selection Process	Methods 2.3	Screening stages described; PRISMA flow diagram provided (Figure 1)	✅
Data Collection Process	Methods 2.3	Extracted variables clearly listed	✅
Data Items	Methods 2.3	Prevalence, anatomical location, gender, etiology, imaging modality, root resorption defined	✅
Risk of Bias Assessment	Methods (Risk of Bias subsection)	STROBE-based qualitative appraisal performed	✅
Effect Measures	Results	Prevalence ranges, proportional distributions reported	✅
Synthesis Methods	Methods 2.3	Qualitative synthesis justified due to heterogeneity	✅
Study Selection Results	Results Section 3	Flow diagram with numerical breakdown (612 → 31 studies)	✅
Study Characteristics	Table 3	Study categories and principal findings summarized	✅
Risk of Bias Results	Integrated in Results & Discussion	Methodological strengths and weaknesses narratively reported	✅
Results of Individual Studies	Section 3	Prevalence ranges and positional patterns detailed	✅
Results of Syntheses	Sections 3–4	Thematic synthesis of epidemiological and etiological findings	✅
Reporting Bias	Discussion 4.7	Acknowledged heterogeneity and potential publication bias	✅
Certainty of Evidence	Discussion 4.7	Confidence in evidence addressed narratively (no GRADE due to heterogeneity)	✅
Limitations	Section 4.7	Study design variability, retrospective bias, diagnostic inconsistencies discussed	✅
Implications	Sections 4.6 & 5	Clinical and diagnostic implications clearly stated	✅
Funding	Funding Statement	Explicit declaration of no external funding	✅

PROSPERO Registration Summary

Review Title

Incidence, Distribution Patterns, and Etiological Determinants of Permanent Canine Impaction: A Systematic Review of Epidemiological, Anatomical, Genetic, and Radiographic Evidence

Review Question

What is the prevalence, anatomical distribution, etiological determinants, and diagnostic imaging characteristics associated with permanent canine impaction in human populations?

Objectives

To systematically synthesize contemporary evidence regarding:

1. The global prevalence of permanent canine impaction
2. Positional distribution patterns (palatal, buccal, mid-alveolar)
3. Etiological determinants (local, skeletal, genetic factors)
4. Root resorption associated with impacted canines
5. Diagnostic accuracy of imaging modalities, particularly CBCT

Population

Human subjects diagnosed with permanent canine impaction (maxillary or mandibular), regardless of age or sex.

Condition/Exposure

Permanent canine impaction diagnosed clinically and/or radiographically.

Outcomes

Primary outcomes:

- Prevalence rates of permanent canine impaction
- Anatomical location of impaction

Secondary outcomes:

- Etiological factors
- Root resorption of adjacent teeth
- Imaging modality performance

Study Designs Included

- Cross-sectional studies
- Retrospective radiographic analyses
- Epidemiological prevalence studies
- Observational etiological investigations

Excluded:

- Case reports
- Small case series

- Syndromic-only studies
- Studies focusing exclusively on third molars

Search Strategy

Databases searched:
- PubMed
- PubMed Central
- ScienceDirect
- Orthodontic specialty journals

Search period:
December 2009 – February 2024
Keywords included:
"canine impaction," "impacted maxillary canine," "mandibular canine impaction," "transmigration," "prevalence," "etiology," "cone-beam computed tomography."

Data Extraction

Extracted variables:
- Sample size
- Prevalence rates
- Anatomical position
- Gender distribution
- Etiological factors
- Imaging modality
- Presence of root resorption

Risk of Bias Assessment

Methodological quality assessed using STROBE-based criteria appropriate for observational studies.

Data Synthesis

Due to heterogeneity in study design, diagnostic criteria, and outcome reporting, quantitative meta-analysis was not performed. A structured qualitative synthesis was conducted.

Funding

This review received no external funding.

Conflicts of Interest

The authors declare no conflicts of interest.
